# Supplementary material for: Loss of a co-twin at birth and subsequent risk of psychiatric disorders
Source: eLife. 2021 Jan 28;10:e63514. doi: 10.7554/eLife.63514 (PMC7843130; doi:10.7554/eLife.63514)
Supplement: Supplementary file 1. — Supplementary Table 1. International Classification of Diseases (ICD), eighth (ICD-8; 1969–1986), ninth (ICD-9; 1987–1996), and tenth (ICD-10; 1997–2013) revisions codes for diagnoses used in this study Supplementary Table 2. Hazard ratios (HRs) with 95% confidence intervals (CIs) for any psychiatric disorder among twins who lose a co-twin at birth, calculated separately for older and younger siblings of the surviving twins, subgroups by psychiatric disorders among parents during follow-up Supplementary Table 3. Hazard ratios (HRs) with 95% confidence intervals (CIs) for any psychiatric disorder among twins who lose a co-twin at birth, subgrouped by or additionally adjusted for diagnosis of congenital abnormalities and severe somatic diseases during follow-up Supplementary Table 4. Hazard ratios (HRs) with 95% confidence intervals (CIs) for any psychiatric disorder among twins who lost a co-twin within 28 days after birth, derived from different Cox models and by subtypes of psychiatric disorders [file elife-63514-supp1.docx]

Supplemental Table 1 International Classification of Diseases (ICD), eighth (ICD-8; 1969–1986), ninth (ICD-9; 1987–1996), and tenth (ICD-10; 1997–2013) revisions codes for diagnoses used in this study

|  | Specific subtypes | **ICD-8** | **ICD-9** | **ICD-10** |
| --- | --- | --- | --- | --- |
| **All psychiatric disorders** |  | 290-315 | 290-315 | F |
| **Subtypes of psychiatric disorders** |  |  |  |  |
| Neurodevelopmental disorders | Attention-deficit hyperactivity disorder | - | 314 | F90 |
|  | Autism spectrum disorder | – | 299 | F84 |
|  | Intellectual disability | 310-315 | 317-319 | F70-F79 |
| Emotional disorders | Mood disorders, *mainly including depressive disorder and bipolar disorder* | 296, 300.4 | 296, 300E, 311 | F30-F39 |
|  | Anxiety disorders | 300.0, 300.2 | 300A, 300C | F40-F41 |
|  | Stress-related disorder | 307, 308.4 | 308, 309 | F43 |
|  | Behavioral and emotional disorders with onset usually occurring in childhood and adolescence | - | 312, 313 | F91-F98 |
| Other psychiatric disorders |  | 290–315, but not in the any of above categories | 290–315, but not in the any of above categories | F, but not in the any of above categories |
| **Congenital malformations, deformations and chromosomal abnormalities** | | 740-759 | 740-759 | Q |
| **Severe somatic diseases** | Myocardial infarction | 410 | 410,412 | I21, I22, I25.2 |
|  | Congestive heart failure | 427.00-427.27, 428.99 | 428 | I50 |
|  | Cerebrovascular disease | 430-438 | 430-438 | G45, G46, I60-I69 |
|  | Chronic pulmonary disease | 460-466, 490-493, 502-508 | 490-496 | J40-J47 |
|  | Connective tissue disease | 710-718 | 710A, 710B, 710E, 714A, 714B, 714C, 714W,714X, 725 | M05, M06, M32-M34, M35.1, M35.3 |
|  | Diabetes | 250.00 | 250 | E10-E14 |
|  | Renal diseases | 580-584, 590 | 582,583 | N01, N03, N05.2-N05.7 |
|  | Liver diseases | 570-576 | 571C, 571E,571F, 571G, 572C, 572D, 572E, 572W, 456A, 456B, 456C | K70.2-K70.4, K71.7, K72.1, K72.9, K73, K74, K76.6, K76.7 |
|  | Ulcer diseases | 530.93, 531-534 | 531-534 | K25-K28 |
|  | HIV infection/AIDS | - | 042-044 | B20-B24 |

Supplementary Table 2 Hazard ratios (HRs) with 95% confidence intervals (CIs) for any psychiatric disorder among twins who lose a co-twin at birth, calculated separately for older and younger siblings of the surviving twins, sub-groups by psychiatric disorders among parents during follow-up

|  | **Population-based matched cohort** | | | | **Twin-sibling family cohort** | | |
| --- | --- | --- | --- | --- | --- | --- | --- |
|  | Number of cases (Crude incidence rate, per 1000 person years), exposed **twins**/unexposed **twins** | HR(95% CI)^*^ | Number of cases (Crude incidence rate, per 1000 person years), exposed **twins** /matched **singletons** | HR(95% CI)^*^ | Number of cases (Crude incidence rate, per 1000 person years), exposed **twins**/**full siblings** | HR(95% CI) ^†^ |  |
| ***By birth order of the full siblings*** |  |  |  |  |  |  |  |
| Surviving twins vs. their older siblings | - | - | - | - | 61(11.92)/50(6.97) | 1.74 (0.54-5.59) |  |
| Surviving twins vs. their younger siblings | - | - | - | - | 90(12.21)/80(9.15) | 1.27 (0.48-3.34) |  |
| ***By psychiatric disorders among parents during follow-up*** |  |  |  |  |  |  |  |
| Yes | 29(9.78)/104(6.34) | 1.36 (0.71-2.61) | 29(9.78)/139(8.24) | 1.83 (0.88-3.80) | 22(11.63)/26(8.66) | 1.26 (0.17-9.38) |  |
| No | 149(12.67)/496(8.14) | 1.53 (1.24-1.87) | 149(12.67)/584(9.65) | 1.42 (1.16-1.73) | 108(12.48)/104(8.06) | 1.74 (0.85-3.58) |  |
| ***By diagnosis of congenital abnormalities*** |  |  |  |  |  |  |  |
| Yes | 54(20.42)/165(14.64) | 1.62 (1.03-2.55) | 54(20.42)/174(14.83) | 2.54 (1.45-4.45) | 40(21.13)/8(8.52) | - |  |
| No | 124(10.26)/435(6.58) | 1.63 (1.30-2.03) | 124(10.26)/549(8.36) | 1.33 (1.07-1.65) | 90(10.40)/122(8.15) | 1.49 (0.71-3.14) |  |
| ***By the presence of severe somatic diseases during follow-up*** |  |  |  |  |  |  |  |
| Yes | 46(14.89)/90(9.52) | 1.88 (0.82-4.30) | 46(14.71)/170(12.79) | 1.41 (0.78-2.54) | 31(13.84)/16(9.17) | - |  |
| No | 132(11.34)/510(7.51) | 1.58 (1.27-1.95) | 132(11.38)/553(8.62) | 1.40 (1.13-1.73) | 99(11.92)/114(8.05) | 1.26 (0.59-2.66) |  |
| ***Additionally adjusted for diagnosis of congenital abnormalities and severe somatic diseases during follow-up*** | 178(12.08)/600(7.76) | 1.53(1.28-1.84) | 178(12.08)/723(9.33) | 1.39(1.17-1.66) | 130(12.32)/130(8.17) | 1.26(0.69-2.31) |  |

^*^ Cox regression models were stratified by matching identifiers (sex, birth year, and gestational age), and adjusted for birth weight for gestational age, maternal age at childbirth, low Apgar score ≤7 at 5/10 min, maternal education level at childbirth, maternal cohabitation status during pregnancy, and family history of psychiatric disorder.

^†^ Cox regression models were stratified by matching identifiers (sex, birth year, gestational age, birth weight for gestational age, birth order), and adjusted for maternal age at childbirth, low Apgar score ≤7 at 5/10 min, maternal education level at childbirth, maternal cohabitation status during pregnancy, and family history of psychiatric disorder.

Supplementary Table 3 Hazard ratios (HRs) with 95% confidence intervals (CIs) for any psychiatric disorder among twins who lose a co-twin at birth, sub-grouped by or additionally adjusted for diagnosis of congenital abnormalities and severe somatic diseases during follow-up

|  | **Population-based matched cohort** | | | | **Twin-sibling family cohort** | | |
| --- | --- | --- | --- | --- | --- | --- | --- |
|  | Number of cases (Crude incidence rate, per 1000 person years), exposed **twins**/unexposed **twins** | HR(95% CI)^*^ | Number of cases (Crude incidence rate, per 1000 person years), exposed **twins** /matched **singletons** | HR(95% CI)^*^ | Number of cases (Crude incidence rate, per 1000 person years), exposed **twins**/**full siblings** | HR(95% CI) ^†^ |  |
| ***By diagnosis of congenital abnormalities*** |  |  |  |  |  |  |  |
| Yes | 54(20.42)/165(14.64) | 1.62 (1.03-2.55) | 54(20.42)/174(14.83) | 2.54 (1.45-4.45) | 40(21.13)/8(8.52) | - |  |
| No | 124(10.26)/435(6.58) | 1.63 (1.30-2.03) | 124(10.26)/549(8.36) | 1.33 (1.07-1.65) | 90(10.40)/122(8.15) | 1.49 (0.71-3.14) |  |
| ***By the presence of severe somatic diseases during follow-up*** |  |  |  |  |  |  |  |
| Yes | 46(14.89)/90(9.52) | 1.88 (0.82-4.30) | 46(14.71)/170(12.79) | 1.41 (0.78-2.54) | 31(13.84)/16(9.17) | - |  |
| No | 132(11.34)/510(7.51) | 1.58 (1.27-1.95) | 132(11.38)/553(8.62) | 1.40 (1.13-1.73) | 99(11.92)/114(8.05) | 1.26 (0.59-2.66) |  |
| ***Additionally adjusted for diagnosis of*** ***congenital*** ***abnormalities and severe somatic diseases during follow-up*** | 178(12.08)/600(7.76) | 1.53(1.28-1.84) | 178(12.08)/723(9.33) | 1.39(1.17-1.66) | 130(12.32)/130(8.17) | 1.26(0.69-2.31) |  |

^*^ Cox regression models were stratified by matching identifiers (sex, birth year, and gestational age), and adjusted for birth weight for gestational age, maternal age at childbirth, low Apgar score ≤7 at 5/10 min, maternal education level at childbirth, maternal cohabitation status during pregnancy, and family history of psychiatric disorder.

^†^ Cox regression models were stratified by matching identifiers (sex, birth year, gestational age, birth weight for gestational age, birth order), and adjusted for maternal age at childbirth, low Apgar score ≤7 at 5/10 min, maternal education level at childbirth, maternal cohabitation status during pregnancy, and family history of psychiatric disorder.

Supplementary Table 4 Hazard ratios (HRs) with 95% confidence intervals (CIs) for any psychiatric disorder among twins who lost a co-twin **within 28 days after birth**, derived from different Cox models and by subtypes of psychiatric disorders

|  | **Population-based matched cohort** | | | | | **Twin-sibling family cohort** | |
| --- | --- | --- | --- | --- | --- | --- | --- |
|  | Number of cases (Crude incidence rate, per 1000 person years), exposed **twins**/unexposed **twins** | HR(95% CI)^*^ | Number of cases (Crude incidence rate, per 1000 person years), exposed **twins** /matched **singletons** | HR(95% CI)^*^ | Number of cases (Crude incidence rate, per 1000 person years), **exposed twins/full siblings** | | HR(95% CI)^*^ |
| **Model information:**  **Model 1**  Controlled for attained age, (as underlying time scale), sex and all prenatal factors (i.e., GA, birth weight for GA maternal age at birth) | 159(12.00)/549(7.92) | 1.55 (1.28-1.87) | 159(12.00)/658(9.47) | 1.38 (1.15-1.66) | 117(12.29)/106(7.56) | | 1.81 (0.92-3.57) |
| **Model 2**  above + neonatal factors (Apgar score) |  | 1.52 (1.26-1.84) |  | 1.34 (1.11-1.60) |  |  | 1.74 (0.88-3.45) |
| **Model 3**  above + family history of psychiatric disorder, education level (mother); cohabitation status (mother) |  | 1.52 (1.25-1.84) |  | 1.38 (1.15-1.67) |  |  | - |
| ***Full adjusted HRs***^†^ ***for subtypes of psychiatric disorders*** |  |  |  |  |  | |  |
| Neurodevelopment disorders (including ADHD, ASD, and intellectual disabilities ) | 67(4.76)/209(2.91) | 1.56 (1.16-2.12) | 67(4.76)/256 (3.53) | 1.44 (1.08-1.93) | 50(4.91)/26(1.78) | | 0.25 (0.02-2.91) |
| Emotional disorders (including depression, anxiety, stress-related disorder) | 94(6.73)/262(3.66) | 1.90 (1.46-2.46) | 94(6.73)/346 (4.82) | 1.57 (1.23-2.01) | 71(7.03)/71(4.96) | | 1.79 (0.76-4.23) |
| Other psychiatric disorders | 69(4.95)/280(3.95) | 1.17 (0.88-1.55) | 69(4.95)/327(4.58) | 1.15 (0.87-1.51) | 49(4.87)/47(3.26) | | 1.47 (0.50-4.37) |

GA, gestational age.

**^*^**Cox regression models were stratified by matching identifiers or family identifier, and adjusted for covariates mentioned in the ‘model information’ column. Attained age was applied as the underlying time scale.

^†^HRs were derived from fully adjusted Cox regression models, i.e., model 3.
