# Supplementary material for: Loss of a co-twin at birth and subsequent risk of psychiatric disorders
Source: eLife. 2021 Jan 28;10:e63514. doi: 10.7554/eLife.63514 (PMC7843130; doi:10.7554/eLife.63514)
Supplement: Transparent reporting form [file elife-63514-transrepform.pdf]

eLIFE
1st Floor
24 Hills Road
Cambridge CB2 1JP, UK
P 01223 855340
W elifesciences.org
T @elife

eLife's transparent reporting form

We encourage authors to provide detailed information *within their submission* to facilitate the interpretation and replication of experiments. Authors can upload supporting documentation to indicate the use of appropriate reporting guidelines for health-related research (see EQUATOR Network), life science research (see the BioSharing Information Resource), or the ARRIVE guidelines for reporting work involving animal research. Where applicable, authors should refer to any relevant reporting standards documents in this form.

If you have any questions, please consult our Journal Policies and/or contact us: editorial@elifesciences.org.

Sample-size estimation

- You should state whether an appropriate sample size was computed when the study was being designed
- You should state the statistical method of sample size computation and any required assumptions
- If no explicit power analysis was used, you should describe how you decided what sample (replicate) size (number) to use

Please outline where this information can be found within the submission (e.g., sections or figure legends), or explain why this information doesn't apply to your submission:

From the Total Population Register, we identified all individuals born in Sweden between 1973 and 2013. As shown in Figure 1, among a total of the 167,600 identified twin individuals, 787 experienced loss of a co-twin at birth, defined as a death of the co-twin within 60 days after birth, and thereby were included in the exposed cohort. The first reference group included 3,935 sex-, birth year-, and gestational age (GA)-matched unexposed twins randomly selected from the Swedish twin population that did not experience such a loss, to control for twin pregnancy and twin birth. Because the exposed twins grew up on their own, as singletons, we also included 3,935 singletons randomly selected from the singleton population that were individually matched to the exposed twins on sex, birth year, and birth factors (GA, birth weight for GA, and birth order) as the second reference group. In addition to the population-based matched cohort, to address the concern of familial confounding, we compared 569 exposed twins to their full siblings (n=880) in twin-sibling family cohort. The study entry was the 60th day after birth of the exposed twin (i.e., the index date).
Since we have used all eligible individuals in Sweden and reached a fairly large sample size, we concluded that we had sufficient power for the planned analyses.

Replicates

- You should report how often each experiment was performed
- You should include a definition of biological versus technical replication
- The data obtained should be provided and sufficient information should be provided to indicate the number of independent biological and/or technical replicates
- If you encountered any outliers, you should describe how these were handled
- Criteria for exclusion/inclusion of data should be clearly stated
- High-throughput sequence data should be uploaded before submission, with a private link for reviewers provided (these are available from both GEO and ArrayExpress)

Please outline where this information can be found within the submission (e.g., sections or figure legends), or explain why this information doesn't apply to your submission:

Given the unique data from national registers, it is indeed challenging to find a replication setting. However, we have performed analyses using three different reference groups (i.e., matched unexposed twin, matched singleton, and the full siblings of exposed twins), and our finding on the increased risk of psychiatric disorders among the surviving twins gains support from the real experience shared by these twinless twins (https://www.twinlesstwins.org/resources/early-twin-loss/).

eLife Sciences Publications, Ltd is a limited liability non-profit non-stock corporation incorporated in the State of Delaware, USA, with company number 5030732, and is registered in the UK with company number FC030576 and branch number BR015634 at the address 1st Floor, 24 Hills Road, Cambridge CB2 1JP | August 2014

Statistical reporting

- Statistical analysis methods should be described and justified
- Raw data should be presented in figures whenever informative to do so (typically when N per group is less than 10)
- For each experiment, you should identify the statistical tests used, exact values of N, definitions of center, methods of multiple test correction, and dispersion and precision measures (e.g., mean, median, SD, SEM, confidence intervals; and, for the major substantive results, a measure of effect size (e.g., Pearson's r, Cohen's d)
- Report exact p-values wherever possible alongside the summary statistics and 95% confidence intervals. These should be reported for all key questions and not only when the p-value is less than 0.05.

Please outline where this information can be found within the submission (e.g., sections or figure legends), or explain why this information doesn't apply to your submission:

The statistical analysis has been thoroughly described in the Methods (pages 12-13).

(For large datasets, or papers with a very large number of statistical tests, you may upload a single table file with tests, Ns, etc., with reference to sections in the manuscript.)

Group allocation

- Indicate how samples were allocated into experimental groups (in the case of clinical studies, please specify allocation to treatment method); if randomization was used, please also state if restricted randomization was applied
- Indicate if masking was used during group allocation, data collection and/or data analysis

Please outline where this information can be found within the submission (e.g., sections or figure legends), or explain why this information doesn't apply to your submission:

The ascertainment of exposure and identification of reference group are described in the Methods (pages 10-11).

Additional data files ("source data")

- We encourage you to upload relevant additional data files, such as numerical data that are represented as a graph in a figure, or as a summary table
- Where provided, these should be in the most useful format, and they can be uploaded as "Source data" files linked to a main figure or table
- Include model definition files including the full list of parameters used
- Include code used for data analysis (e.g., R, MatLab)
- Avoid stating that data files are "available upon request"

Please indicate the figures or tables for which source data files have been provided:

The model definition, including covariates, has been thoroughly described in Methods (pages 10-11).
We have uploaded SAS script used for primary analyses.

eLife Sciences Publications, Ltd is a limited liability non-profit non-stock corporation incorporated in the State of Delaware, USA, with company number 5030732, and is registered in the UK with company number FC030576 and branch number BR015634 at the address 1st Floor, 24 Hills Road, Cambridge CB2 1JP | August 2014
